# Progressive loss of muscle mass could be an adverse prognostic factor of 28-day mortality in septic shock patients

**DOI:** 10.1038/s41598-019-52819-w

**Published:** 2019-11-11

**Authors:** Dong-Woo Seo, Kyung Won Kim, Chang Hwan Sohn, Seung Mok Ryoo, Youn-Jung Kim, Ahn Shin, Won Young Kim

**Affiliations:** 1 Department of Biomedical Informatics, University of California San Diego, School of Medicine, La Jolla, USA; 2 Department of Emergency Medicine, University of Ulsan, College of Medicine, Asan Medical Center, Seoul, South Korea; 3 Department of Radiology, University of Ulsan, College of Medicine, Asan Medical Center, Seoul, South Korea

**Keywords:** Infectious diseases, Metabolic disorders

## Abstract

A decrease in skeletal muscle mass has been shown to increase hospital mortality. Nevertheless, little is known about the association between progressive muscle loss over time and clinical outcomes. We aimed to evaluate whether progressive loss of muscle mass in septic shock patients was associated with mortality. We reviewed prospectively enrolled registry of septic shock which had 817 consecutive patients. Of these, 175 patients who had computed tomography (CT) at a time of admission as well as 3–6 months prior to admission were included. Between these two CTs, the change in total abdominal muscle area index (TAMAI) was evaluated for progressive muscle loss. The change in TAMAI was higher in the non-survivors (−7.6 cm^2^/m^2^, 19.0% decrease) than the survivors (−4.0 cm^2^/m^2^, 10.5% decrease) with statistical significance (*p* = 0.002). Multiple logistic regression showed that the patients who had more than a 6.4 cm^2^/m^2^ (16.7%) reduction of TAMAI had a 4.42-fold higher risk for mortality at 28 days (OR, 4.42; 95% CI, 1.41–13.81, *p* = 0.011). Our study suggested that progressive loss of muscle mass might be a useful prognostic factor for septic shock patients. This implication will need to be further explored in future prospective studies.

## Introduction

Sepsis has been one of the most expensive disease conditions treated in the United States. Sepsis resulted in a cost of 5.2% of the total costs for all admissions in 2011^1^. According to the third international definition^2^, sepsis is defined as life-threatening organ dysfunction caused by a dysregulated host response to infection. Septic shock is characterized by persistent hypotension with vasopressors and elevated serum lactate despite adequate volume replacement^2^. Since the Surviving Sepsis Campaign, the in-hospital mortality of sepsis-related conditions appears to be decreasing; nevertheless, it remains still unacceptably high^2^. Intensive care unit (ICU) patients in septic shock often experience muscle catabolism, muscle weakness, and several metabolic dysfunctions that are currently classified as sarcopenia or cachexia^3,4^. In patients with cirrhosis, an association between low muscle mass and sepsis has been reported, but there is controversy about its association with mortality^5–7^. A recent study showed an association of muscle mass loss and increased mortality in elderly sepsis patients^8^.

Sarcopenia is defined as decreased skeletal muscle mass and function leading to the decline of physical ability, and recently classified as a disease from the International Classification of Diseases (ICD-10CM)^9,10^. Sarcopenia was proven to increase physical disability, healthcare costs, adverse cardiometabolic effects and notably increased mortality in various diseases^10,11^. Recently, progressive loss in skeletal muscle has gained emphasis for its stronger prognostic value to predict mortality in cancer patients^12,13^.

In the patients with sepsis or septic shock, there have been a few studies describing an association between sepsis or septic shock and muscle mass^7,8,14^. Nevertheless, to the best of our knowledge, there has been no evidence regarding the effect of progressive loss of muscle mass on sepsis or septic shock. Previous studies analyzed muscle mass using only values at a specific time point. From this perspective, we aim to determine the prognostic value of progressive loss of muscle mass in predicting 28-day mortality outcome in patients with septic shock, using a single-center, observational, prospectively-collected registry.

## Results

### Patients

**Figure 1:**
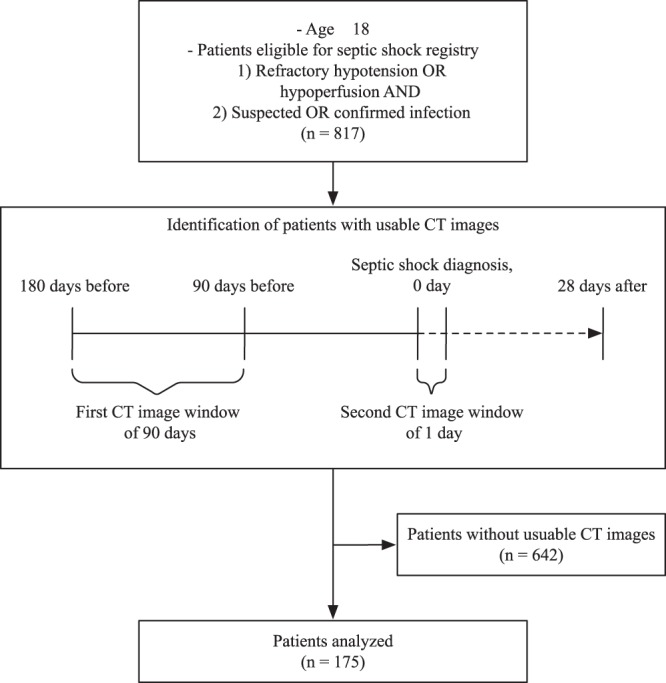
Patient flow diagram showing study timeline and patient selection.

During the study period, a total of 817 patients with septic shock were admitted to the emergency ICU and enrolled in the septic shock registry (Fig. 1). Of these, a total of 175 patients who had two abdominal CT scans for any purpose at 3–6 months before admission (first CT) and at a time of admission (second CT) were selected. In the 175 included patients, there was no missing data regarding study outcomes or the main variables considered in the analysis.

Among study patients, 157 (89.7%) patients survived to the 28-day mark, while 18 (10.3%) patients died within 28 days after diagnosis of septic shock. The baseline characteristics of all patients, survivors, and non-survivors are summarized in Table 1. The median age of all patients was 65.0 years, and 62.9% were male. There were no significant differences in terms of past medical history between the survivors and non-survivors except for coronary artery disease (7.0% vs. 22.2%, p = 0.029). There was a difference between survivors and non-survivors in terms of initial respiratory rate (20.0, interquartile ranges [IQR] 20.0 to 20.0 vs. 24.0, IQR 20.0 to 24.0, p < 0.001) and lactate (2.7, IQR 1.9 to 4.4 vs. 4.3, IQR 3.0 to 7.4, p < 0.003). The SOFA (sequential organ failure assessment) score of the non-survival group was higher than that of the survival group (9.5, IQR 7.2 to 11.5 vs. 7.0, IQR 5.0 to 9.0, p = 0.005).

**Table 1:** Baseline characteristics of the study patients according to 28-day mortality.

	Total (n = 175)	Survivor (n = 157)	Non-Survivor (n = 18)	p-value
Age (year)	65.0 (58.0–72.0)	65.0 (58.0–72.0)	66.0 (61.2–75.0)	0.423
Gender (Male)	110 (62.9%)	99 (63.1%)	11 (61.1%)	0.871
Hypertension	37 (21.1%)	30 (19.1%)	7 (38.9%)	0.052
Diabetes mellitus	40 (22.9%)	33 (21.0%)	7 (38.9%)	0.087
Coronary artery disease	15 (8.6%)	11 (7.0%)	4 (22.2%)	0.029
Chronic lung disease	6 (3.4%)	4 (2.5%)	2 (11.1%)	0.059
Chronic renal failure	5 (2.9%)	5 (3.2%)	0 (0.0%)	0.442
Liver cirrhosis	37 (21.1%)	35 (22.3%)	2 (11.1%)	0.271
Stroke	6 (3.4%)	4 (2.5%)	2 (11.1%)	0.059
Cancer	112 (64.0%)	99 (63.1%)	13 (72.2%)	0.443
Respiratory rate, initial (/min)	20.0 (20.0–21.5)	20.0 (20.0–20.0)	24.0 (20.0–24.0)	0.001
Lactate, initial (mmol/L)	2.9 (2.0–4.8)	2.7 (1.9–4.4)	4.3 (3.0–7.4)	0.003
SOFA score, at admission	7.0 (5.0–9.0)	7.0 (5.0–9.0)	9.5 (7.2–11.5)	0.005
Respiratory	1.0 (0.0–1.0)	1.0 (0.0–1.0)	1.5 (1.0–3.0)	0.011
Cardiovascular	3.0 (3.0–4.0)	3.0 (3.0–4.0)	4.0 (3.0–4.0)	0.298
Renal	0.0 (0.0–1.0)	0.0 (0.0–1.0)	0.5 (0.0–2.0)	0.309
Coagulation	1.0 (0.0–2.0)	1.0 (0.0–2.0)	2.0 (0.2–2.8)	0.097
Hepatic	1.0 (0.0–2.0)	1.0 (0.0–2.0)	0.0 (0.0–1.0)	0.184
Neurologic	0.0 (0.0–0.0)	0.0 (0.0–0.0)	0.0 (0.0–0.0)	0.016
Height, at admission (cm)	162.2 (155.0–168.8)	162.0 (155.0–168.8)	163.9 (152.9–168.4)	0.696
Weight, at admission (kg)	56.2 (50.5–64.0)	57.0 (51.0–64.0)	54.5 (46.2–63.5)	0.378
BMI, at admission (kg/m^2^)	21.8 (19.6–24.0)	21.9 (19.7–24.0)	20.9 (19.4–24.1)	0.356
Low TAMAI, at admission	151 (86.3%)	136 (86.6%)	15 (83.3%)	0.701
TAMAI, at a previous visit (cm^2^/m^2^)	38.3 ± 8.5	38.1 ± 8.0	39.9 ± 11.7	0.405
TAMAI, at admission (cm^2^/m^2^)	34.0 ± 8.4	34.2 ± 8.3	32.3 ± 8.5	0.377
TAMAI difference, numeric (cm^2^/m^2^)	4.3 ± 4.6	4.0 ± 4.3	7.6 ± 6.3	0.002
TAMAI difference, dichotomous, >6.4 cm^2^/m^2^	55 (31.4%)	44 (28.0%)	11 (61.1%)	0.004

Values are presented as median with interquartile range or number (percent).

SOFA, Sequential Organ Failure Assessment; BMI, Body Mass Index; TAMAI, Total Abdominal Muscle Area Index.

In terms of body metrics, including height, weight, and BMI, there were no significant differences. The overall prevalence of low TAMAI (total abdominal muscle area index) at the time of admission for septic shock was 86.3% (151 out of 175), and there was no difference between the survival and non-survival groups (86.6% vs. 83.3%, p = 0.701). Among the four BMI categories, the normal weight group was most common (n = 93), followed by underweight group (n = 55), overweight group (n = 24), and obese group (n = 3) (Supplementary Table 1). Low TAMAI was prevalent in all BMI categories (87.3% in underweight, 81.7% in normal weight, 100% in overweight, and 100% in obese). There were no significant differences between the survival and non-survival groups in each BMI category (Supplementary Table 1).

**Figure 2:**
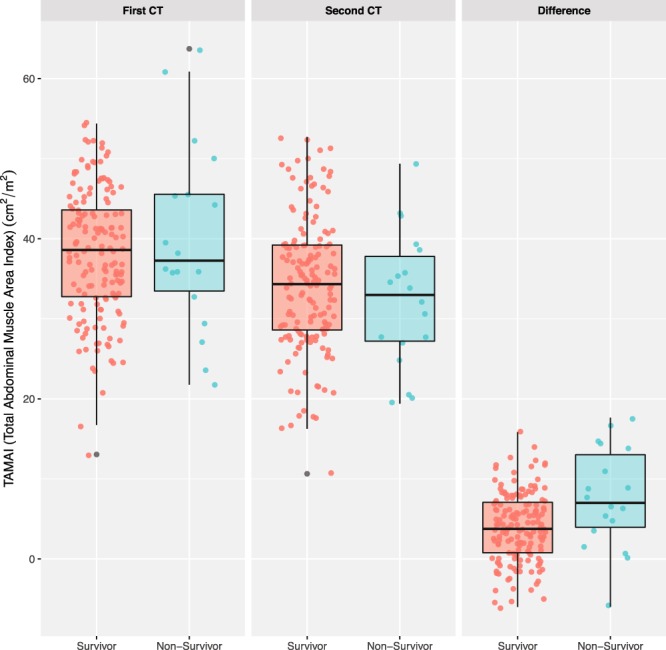
Distribution of skeletal muscle index on CT and 28-day mortality. The TAMAI difference was calculated by subtracting the TAMAI value of the second CT from that of the first CT.

Between survivors and non-survivors, TAMAI values did not differ at the time of admission (34.2 cm^2^/m^2^ vs. 32.3 cm^2^/m^2^, p = 0.377) as well as at the previous visit (38.1 cm^2^/m^2^ vs. 39.9 cm^2^/m^2^, p = 0.405). When comparing TAMAI values between the two-time points, TAMAI decreased by 19.0% in the non-survivors, while it decreased by 10.5% in the survivors (Fig. 2). These changes in TAMAI values differed significantly between non-survivors and survivors (7.6 cm^2^/m^2^ vs. 4.0 cm^2^/m^2^, p = 0.002).

**Figure 3:**
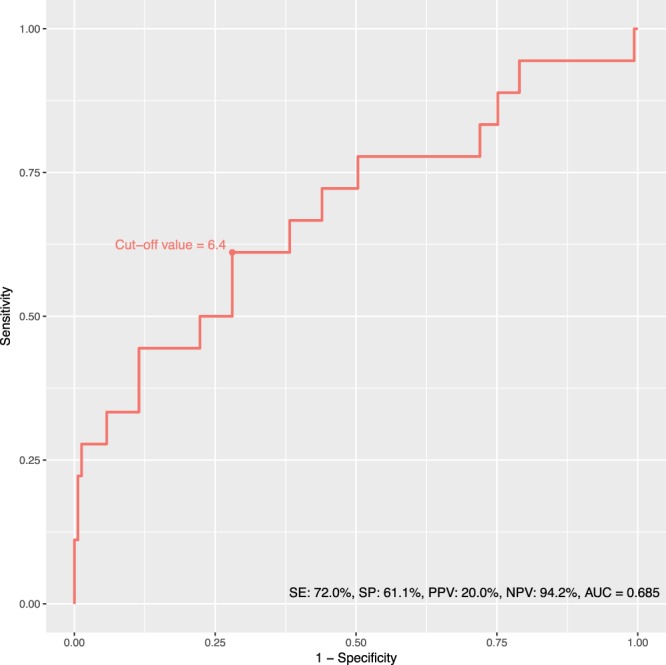
Receiver operating characteristic (ROC) curves for the difference of total abdominal muscle area index (TAMAI) to predict 28-day mortality in sepsis patients.

Receiver operating characteristic analysis demonstrated that the TAMAI could predict 28-day mortality with good discriminative power (AUC 0.685), as illustrated in Fig. 3. The optimal cut-off values of TAMAI to predict 28-day mortality was determined as 6.4 cm^2^/m^2^ (16.7% reduction of TAMAI) based on Youden’s index. The TAMAI reduction with the cut-off value of 6.4 cm^2^/m^2^ predicted 28-day mortality with a sensitivity of 72.0%, a specificity of 61.1%, a positive predictive value of 20% and a negative predictive value of 94.2%.

**Figure 4:**
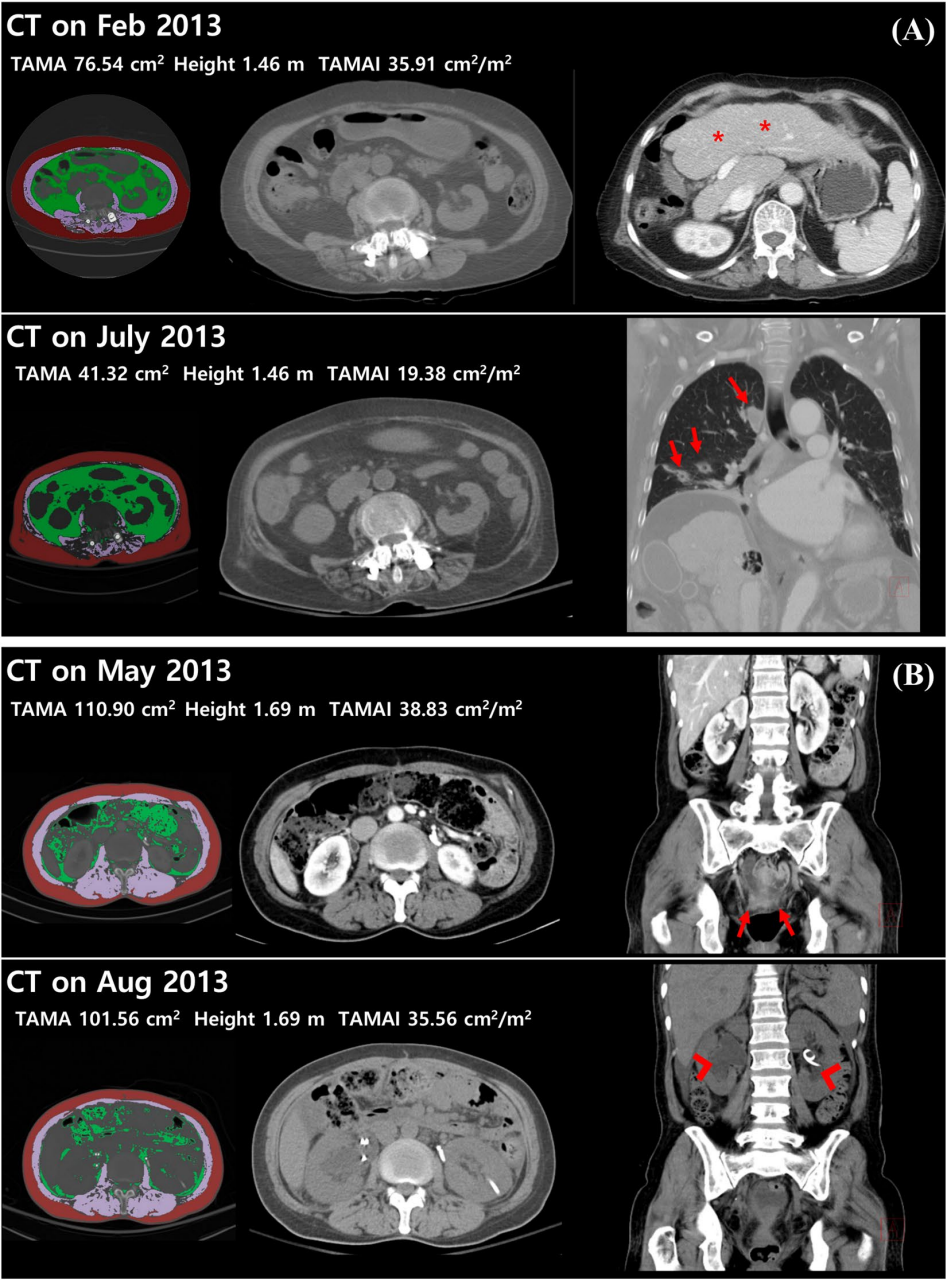
Representative cases with and without progressive sarcopenia. (**A**) A 70 year old female with progressive sarcopenia. During treatment of biliary liver cirrhosis (asterisks) with steroid, sarcopenia was aggravated (TAMAI; 35.91 on Feb 2013 and 19.38 on July 2013). On July 2013, pneumonia occurred due to septic embolism (arrows). She was admitted to intensive care unit and treated with antibiotics. However, she was expired after 2 days. (**B**) A 54 year old female without progressive sarcopenia. During chemotherapy for stomach cancer with peritoneal carcinomatosis (arrows), the muscle mass was not changed (TAMAI; 38.83 on May 2013 and 35.56 on Aug 2013). On Aug 2013, sepsis shock occurred due to urosepsis with hydronephrosis (arrowheads). She was admitted to intensive care unit and treated with double-J stent insertion, percutaneous nephrostomy, and antibiotics. After 10 days, she was recovered.

The results of multiple logistic regression test are presented in Table 2. Coronary artery disease, respiratory rate, lactate and SOFA score were not predictive of 28-day mortality. The difference of TAMAI using the optimal cut-off value (6.4 cm^2^/m^2^) was the only independent predictor for 28-day mortality (OR, 4.42; 95% CI, 1.41–13.81, p = 0.011), and with high goodness-of-fit of the model (p > 0.05). There was no significant multicollinearity. These results suggested that progressive muscle loss can be used as a prognostic factor to predict 28-day mortality, as illustrated in the representative cases (Fig. 4).

**Table 2:** Adjusted odds ratios for 28-day mortality of sepsis patients.

	Odds Ratio	95% Confidence Interval	p-value
Coronary artery disease	3.755	0.836–16.862	0.084
Respiratory rate, initial (/min)	1.036	0.929–1.154	0.527
Lactate, initial (mmol/L)	1.054	0.871–1.275	0.588
SOFA score, at admission	1.223	1.000–1.497	0.05
TAMAI difference, dichotomous, >6.4 cm^2^/m^2^	4.420	1.414–13.812	0.011
(TAMAI difference, numeric (cm^2^/m^2^))	(1.172)	(1.040–1.322)	(0.009)

SOFA, Sequential Organ Failure Assessment; TAMAI, Total Abdominal Muscle Area Index.

## Discussion

In this study, we found that the progressive loss of skeletal muscle is an adverse prognostic factor for 28-day mortality in septic shock patients. The patients who had more than a 6.4 cm^2^/m^2^ (16.7%) reduction of TAMAI had a 4.42-fold higher risk for mortality at 28 days.

So far, it has been well-known through extensive previous studies that loss of muscle mass can predict poor prognosis in cancer^9,15,16^, transplantation^6,17–19^, ICU-admitted patients^9,14,20–22^ and sepsis patients^7,8,14^. However, the majority of previous reports have used muscle mass measured at one-time point. Only a few studies investigated the clinical impact of progressive muscle loss on patients^12,13^. In our study cohorts, low TAMAI at one-time point either admission or 3–6 months prior to admission did not show prognostic value to predict 28-day mortality, while the progressive muscle loss was identified as a prognostic factor for 28-day mortality in septic shock patients. Our study results might be meaningful to support the emerging concept of progressive loss of muscle mass.

A recent study by Shibahashi *et al*. demonstrated an association of muscle mass loss and increased mortality in elderly sepsis patients^8^. They suggested that the cutoff value of skeletal muscle area to predict mortality was 45.2 cm^2^ for men and 39.0 cm^2^ for women^8^. With these cutoff values, OR for decreased muscle area was 3.27 (95% CI, 1.61–6.63, P = 0.001)^8^. Unlike other studies, including our study, Shibahashi’s work did not compensate for height; therefore, care should be taken in its interpretation^15,16,20,22^. Performing studies in the same settings are difficult because there is no consensus regarding the cutoff value of sarcopenia. This value might be affected by confounding factors such as age, race and a specific disease. Weijs *et al*. reported that decreased muscle mass, as assessed by CT cross sectionally, was an independent risk factor for mortality in mechanically ventilated critically ill patients^21^. The OR for mortality of Weijs’s study was 4.3 (95% CI, 2.0–9.0, p < 0.011), and was similar to our result for TAMAI difference (OR, 4.42; 95% CI, 1.41–13.81, p = 0.011) (Table 2)^21^. Moisey *et al*. also showed that increased muscle index was significantly associated with decreased mortality in severely injured elderly ICU patients (OR, 0.93, 95% CI: 0.88–1.00, P = 0.025)^20^. Although Moisey’s study used a different cutoff of TAMAI from the one used in our study, given our analysis of the numerical value of TAMAI, the results trended in the same direction^20^. Ji *et al*. reported that decreased muscle mass was an independent risk factor for 30-day mortality in critically ill patients with intra-abdominal sepsis^14^.

Contrary to these previous studies in which decreased muscle mass at a time point was able to predict mortality in patients with sepsis or critically ill conditions, our study did not show prognostic value of decreased muscle mass at a time of admission. We postulate that this discrepancy might be due to the unique characteristics of our study cohorts. Our hospital is the biggest tertiary referral hospital in our nation especially in cancer, and we have a dedicated emergency unit only for cancer patients. Therefore, 64% of our study cohort was cancer patients in septic shock, and a majority of them had critical or severe conditions even at the prior visit as well as admission for septic shock. These characteristics of our study cohort can explain the overall prevalence of sarcopenia of 83% in our study. This was slightly higher than that of other studies in ICU patients. Peterson *et al*. reported the rate of sarcopenia in ICU patients was much higher than that of other patients^9^. The reported prevalence of sarcopenia in ICU was 60–70%, while that of other diseases such as cancer or liver disease was 15–60% or 30–45%^6,9,15,16,18^.

In terms of pathophysiologic background, skeletal muscle mass has been increasingly recognized as an important proxy for physiologic reserve^23,24^. It is highly important in glucose disposal, protein synthesis and mobility^23,24^. Loss of muscle mass has also been associated with increased infection rates^25^. Increased infection rates in decreased muscle mass patients might be explained by the essential role of skeletal muscle, which activates the immune system in dangerous conditions^26^.

Interestingly, although it is thought that obesity is an adverse prognostic factor in many diseases, there has been much debate regarding this assumption. Despite population-based studies reporting higher mortality, studies of obesity in critical illness patients have yielded mixed results, referred to as the ‘obesity paradox’^27–30^. Indeed, our results showed that BMI was not associated with mortality (Table 1, Supplementary Table 1). Most decreased muscle mass studies reported similar results, suggesting that decreased muscle mass, not BMI, might be a cause associated with mortality^9,14,16,20–22^.

There are several limitations to our study. We included only patients with two CTs that met inclusion criteria. Consequently, only 21.4% of patients were included from the septic shock registry (Supplementary Table 2). Main limitation of the present study is the lack of power (0.65). The low number of non-survivors (n = 18) might have contributed to the lack in significance of some results. Hence, the results of our study should be interpreted with the limitation of under-powered and over-interpretation should be avoided. There might be selection bias, because chronically ill patients with frequent CT follow-up might have a higher chance to be included than acutely ill patients without prior CT. The definition of sarcopenia requires both muscle mass index and function, but our study could not include the assessments of muscle strength or physical performance. Although the skeletal muscle mass assessed with CT reportedly correlates well with muscle strength and physical performance^31^, this was another limitation. In addition, we could not collect specific infection sources, causes of death or weight at the previous visit. This study was performed at a single center which has a unique characteristic of the tertiary referral center with a dedicated emergency unit for cancer; therefore, generalizability may be limited. However, a single center study was enough to investigate the feasibility to use progressive muscle loss as a prognostic factor in septic shock patients, and bridge for the large-scale multi-center study in the near future. In summary, our study suggested that the progressive loss of skeletal muscle may have a promising role as an early prognostic factor for septic shock patients. Future large-scale multi-center studies will be needed to clarify our results and proposals.

## Methods

### Study design

Our institutional review board approved the study and waived the requirement for informed consent (IRB Approval Number S2017-0995-0002). This study is reported according to the Transparent Reporting of a multivariable prediction model for Individual Prognosis or Diagnosis (TRIPOD) guidelines^32^.

This retrospective, single-center cohort study using a prospective septic shock registry was conducted at the emergency department (ED) of a university-affiliated, tertiary referral center in Seoul, Korea, with an annual census of approximately 110,000 visits^33–35^. The study period was January 1, 2013 to December 31, 2015. Adult (≥18 years) patients admitted to the ED were enrolled in the septic shock registry when they showed evidence of refractory hypotension or hypoperfusion and suspected or confirmed infection^36–38^. As described in the previous study, refractory hypotension was defined as persistent hypotension (systolic blood pressure, <90 mmHg; mean arterial pressure, <70 mmHg; or systolic blood pressure decrease of >40 mmHg) after 20–30 ml/kg or more intravenous fluid challenge, or requiring vasopressors to maintain a systolic blood pressure of ≥90 mmHg or mean arterial pressure of ≥70 mmHg^39^. Hypoperfusion was defined as serum lactate levels of ≥4 mmol/l^39^. Our septic shock registry did not include patients who refused intensive treatment or signed a “Do Not Attempt Resuscitation” order or who refused to enroll in the registry. We included patients who had two abdominal CT images for any purpose. The first CT image was obtained 90–180 days before the septic shock diagnosis and the second CT image was on the day of septic shock diagnosis (Fig. 1).

### Imaging and assessment of muscle mass

Body mass index (BMI) was defined as the weight in kilograms divided by the square of the height in meters (kg/m^2^)^15,40^ and was categorized as underweight (BMI <20.0 kg/m^2^), normal weight (BMI 20.0 to 24.9 kg/m^2^), overweight (BMI 25.0 to 29.9 kg/m^2^), or obese (BMI ≥30.0 kg/m^2^)^15^. Body composition was evaluated using CT images acquired during underlying disease treatment and follow-up. An experienced radiologist (K.W.K), who was blind to clinical information performed the image analysis using AsanJ-Morphometry^TM^ software. This is dedicated software for measuring abdominal muscle and fat area based on ImageJ (NIH, Bethesda, MD, USA)^41^.

As described in the previous work, the inferior endplate level of the L3 vertebra was chosen as a landmark because it correlates with whole-body skeletal muscle mass and adipose-tissue mass^42^. Total abdominal muscle area (TAMA), including all muscles on the selected axial images, *i.e*., psoas, paraspinal, transversus abdominis, rectus abdominis, quadratus lumborum, and internal and external obliques, were demarcated using predetermined thresholds (−29 to +190 Hounsfield units). The visceral fat area (VFA) and the subcutaneous fat area (SFA) were also demarcated using fat tissue thresholds (−190 to −30 Hounsfield units). The TAMA was normalized for the square of the height (expressed in units of cm^2^/m^2^) and was referred to as the TAMA index (TAMAI). According to Martin’s study, in women, low TAMAI was defined as <41 cm^2^/m^2^ regardless of BMI^15^. For men, low TAMAI was defined as <43 cm^2^/m^2^ when BMI was <25 kg/m^2^, and <53 cm^2^/m^2^ when BMI was 25 kg/m^2^ or more^15^. TAMAI difference was calculated by subtracting the TAMAI value of the second CT (CT image at septic shock diagnosis) from that of the first CT (CT image of 90–180 days before septic shock diagnosis).

### Statistical analysis

Continuous variables were expressed as means with standard deviations or median with interquartile ranges (IQR) if they did not satisfy the assumption of a normal distribution. We used the Shapiro-Wilk test for the test of normality. Categorical variables were expressed as numbers and percentages. The Mann-Whitney U test, t-test Fisher’s exact test was used to compare the values of continuous variables. The Chi-square test was used for categorical variables. The prognostic value of the difference of TAMAI was analysed using the receiver operating characteristic (ROC) curves with the area under the cure (AUC). The optimal cut-off value was determined using Youden’s index. To assess the independent contribution of each variable to 28 days mortality, multivariate logistic regression analyses were performed with the inclusion of candidate predictors which were significant at the univariate comparison between survivors and non-survivors. The results of the logistic regression analysis were presented as odds ratios (OR) and 95% confidence intervals (CI). Variables with the significance of p < 0.05 in univariate analysis were selected for multivariable analysis. The backward stepwise elimination method was used to determine the final, significant predictors. Multicollinearity was assessed by inspecting the correlation matrices of independent variables and by calculating the variance inflation factor (VIF). VIF values greater than ten were regarded as indicating serious multicollinearity. To determine the goodness-of-fit of the models, the Hosmer–Lemeshow test was used to assess whether the model differed significantly from a perfect prediction model. For all the analyses, a two-sided P value of <0.05 was considered to indicate a statistically significant difference. Statistical analyses were performed by using R version 3.5.0 (R Foundation for Statistical Computing, Vienna, Austria).

## Supplementary information

Supplementary Tables

## Data Availability

The datasets generated and analysed during the current study are available from the corresponding author on reasonable request.
