# Supplementary material for: Progressive loss of muscle mass could be an adverse prognostic factor of 28-day mortality in septic shock patients
Source: Sci Rep. 2019 Nov 11;9:16471. doi: 10.1038/s41598-019-52819-w (PMC6848164; doi:10.1038/s41598-019-52819-w)
Supplement: Supplementary file 1 — Supplementary Tables [file 41598_2019_52819_MOESM1_ESM.pdf]

Title: Progressive loss of muscle mass could be an adverse prognostic factor of 28-day mortality in septic shock patients

Dong-Woo Seo[a], MD

Department of Biomedical Informatics, University of California San Diego, School of Medicine

Department of Emergency Medicine, University of Ulsan, College of Medicine, Asan Medical Center

Kyung Won Kim[a], MD

Department of Radiology, University of Ulsan, College of Medicine, Asan Medical Center

Chang Hwan Sohn, MD

Department of Emergency Medicine, University of Ulsan, College of Medicine, Asan Medical Center

Seung Mok Ryoo, MD

Department of Emergency Medicine, University of Ulsan, College of Medicine, Asan Medical Center

Youn-Jung Kim, MD

Department of Emergency Medicine, University of Ulsan, College of Medicine, Asan Medical Center

Ahn Shin, MD

Department of Emergency Medicine, University of Ulsan, College of Medicine, Asan Medical Center

Won Young Kim, MD, PhD[*]

Department of Emergency Medicine, University of Ulsan, College of Medicine, Asan Medical Center

Corresponding author

Won Young Kim, MD, PhD[*]

Associate Professor, Department of Emergency Medicine

University of Ulsan, College of Medicine, Asan Medical Center

88, Olympic-ro 43-gil, Songpa-gu, Seoul 05505, Korea

Tel: +82-2-3010-3350; Fax: +82-2-3010-3360; E-mail: wonpia73@gmail.com (mailto:wonpia73@gmail.com)

[a] These authors contributed equally to this work.

[*] Corresponding author

Supplementary Information

Supplementary Table 1. Body mass index, low TAMAI, and 28-day mortality in each BMI category.

	**Underweight** (BMI < 20.0 kg/m^2)				**Normal weight** (BMI 20.0 to 24.9 kg/m^2)				**Overweight** (BMI 25.0 to 29.9 kg/m^2)				**Obese** (BMI ≥ 30.0 kg/m^2)			
	**Survival**	**Non-Survival**	**Subtotal**	**p-value**	**Survival**	**Non-Survival**	**Subtotal**	**p-value**	**Survival**	**Non-Survival**	**Subtotal**	**p-value**	**Survival**	**Non-Survival**	**Subtotal**	**p-value**
**Normal skeletal muscle**	6 (12.5%)	1 (14.3%)	7 (12.7%)	0.895	15 (17.9%)	2 (22.2%)	17 (18.3%)	0.747	0 (0%)	0 (0%)	0 (0%)	NA	0 (0%)	0 (0%)	0 (0%)	NA
**Low TAMAI**	42 (87.5%)	6 (85.7%)	48 (87.3%)		69 (82.1%)	7 (77.8%)	76 (81.7%)		22 (100%)	2 (100%)	24 (100%)		3 (100%)	0 (0%)	3 (100%)	
**Subtotal**	48 (100%)	7 (100%)	55 (100%)		84 (100%)	9 (100%)	93 (100%)		22 (100%)	2 (100%)	24 (100%)		3 (100%)	0 (0%)	3 (100%)	

Supplementary Table 2. Baseline characteristics of included and not included patients.

	Total (n = 817)	One or less CT, not included group (n = 642)	Two CT, included group (n = 175)	p-value
**Age**	66.0 (58.0-74.0)	67.0 (58.0-74.0)	65.0 (58.0-72.0)	0.263
**Respiratory rate, initial (/min)**	20.0 (20.0-24.0)	20.0 (20.0-24.0)	20.0 (20.0-21.5)	0.000
**SOFA score, at admission**	7.0 (5.0-10.0)	8.0 (5.0-10.0)	7.0 (5.0-9.0)	0.148
**Respiratory**	1.0 (0.0-2.0)	1.0 (0.0-2.0)	1.0 (0.0-1.0)	0.002
**Cardiovascular**	3.0 (3.0-4.0)	4.0 (3.0-4.0)	3.0 (3.0-4.0)	0.100
**Renal**	1.0 (0.0-2.0)	1.0 (0.0-2.0)	0.0 (0.0-1.0)	0.004
**Coagulation**	1.0 (0.0-2.0)	0.0 (0.0-2.0)	1.0 (0.0-2.0)	0.074
**Hepatic**	0.0 (0.0-2.0)	0.0 (0.0-1.0)	1.0 (0.0-2.0)	0.000
**Neurologic**	0.0 (0.0-0.0)	0.0 (0.0-0.0)	0.0 (0.0-0.0)	0.001
**Lactate, initial (mmol/L)**	3.0 (2.0-4.7)	3.0 (2.0-4.7)	2.9 (2.0-4.8)	0.894
**Sex (Male)**	480 (58.8%)	370 (57.6%)	110 (62.9%)	0.213
**Hypertension**	250 (30.6%)	213 (33.2%)	37 (21.1%)	0.002
**Stroke**	57 (7.0%)	51 (7.9%)	6 (3.4%)	0.038
**Diabetes mellitus**	208 (25.5%)	168 (26.2%)	40 (22.9%)	0.367
**Coronary artery disease**	94 (11.5%)	79 (12.3%)	15 (8.6%)	0.170
**Chronic lung disease**	71 (8.7%)	65 (10.1%)	169 (96.6%)	0.018
**Cancer**	307 (37.6%)	195 (30.4%)	112 (64.0%)	0.000
**Chronic renal failure**	53 (6.5%)	48 (7.5%)	5 (2.9%)	0.028
**Liver cirrhosis**	101 (12.4%)	64 (10.0%)	37 (21.1%)	0.000
**28-day mortality**	147 (18.0%)	129 (20.1%)	18 (10.3%)	0.003
